# Postoperative intussusception in children operated for primary intussusception - A very rare entity

**DOI:** 10.12669/pjms.40.5.8276

**Published:** 2024

**Authors:** Muhammad Amjad Chaudhary, Chandni, Aisha Jamil, Sadia Asmat Burki

**Affiliations:** 1 Muhammad Amjad Chaudhary, MS Pediatric Surgery, FRCS, FRCS, Department of Neonatal and Pediatric Surgery, The Children Hospital, Pakistan Institute of Medical Sciences, SZAB Medical University, Islamabad, Pakistan; 2 Chandni,, Department of Neonatal and Pediatric Surgery, The Children Hospital, Pakistan Institute of Medical Sciences, SZAB Medical University, Islamabad, Pakistan; 3 Aisha Jamil, FCPS Pediatric Surgery, Department of Neonatal and Pediatric Surgery, The Children Hospital, Pakistan Institute of Medical Sciences, SZAB Medical University, Islamabad, Pakistan; 4 Sadia Asmat Burki, FCPS Pediatric Surgery, Department of Neonatal and Pediatric Surgery, The Children Hospital, Pakistan Institute of Medical Sciences, SZAB Medical University, Islamabad, Pakistan

**Keywords:** Postoperative intussusception, Small bowel intussusception, Ileocolic, Ileoileal, Abdominal ultrasound

## Abstract

Postoperative intussusception (POI) after abdominal and non-abdominal operations is a rare but recognized condition discussed several times in literature. There are scarce reports regarding POI in children operated primarily for intussusception. We discuss three such cases that were seen in our institution in the last two years. The patients showed symptoms of atypical ileus that failed to resolve two to eight days following primary surgery. Ultrasound reported intussusception and surgical intervention was sought. All patients had ileoileal intussusception. Manual reduction was successful in two cases. One had intestinal necrosis and underwent resection and anastomosis. Recovery was satisfactory without recurrence. POI should be suspected in patients who show signs of intestinal obstruction in early postoperative period. A second POI should be kept in mind after surgical reduction of the first intussusception. Ultrasound should be performed to aid diagnosis followed by urgent surgical intervention.

## INTRODUCTION

Intestinal obstruction is a well-recognized postoperative complication after abdominal operations and is usually attributed to paralytic ileus (adynamic) or adhesions. In the paediatric surgery patient, a recognized but frequently overlooked cause of intestinal obstruction is postoperative intussusception (POI).^1^ The reported incidence of POI after laparotomies is 0.01-0.25%^2^ but it can also occur in non-abdominal surgeries.^3^ Low incidence is the reason why it is often overlooked in the differential diagnosis of postoperative intestinal obstruction. POI has been discussed many times in literature^3^, however very little is reported about POI in patients operated primarily for intussusception.^4^ To increase awareness about this forgotten entity and the potential morbidity associated with it, we present three cases of ileoileal postoperative intussusceptions after primary ileocolic intussusceptions that were seen in our institution over the last two years.

## CASE SERIES

One hundred and sixty four cases of intussusception between the ages of three months to twelve years were managed from May 2021 to April 2023 in the Department of Pediatric Surgery, Pakistan Institute of Medical Sciences, among which postoperative intussusception was seen in three patients.

### Case-1

**Fig.1:**
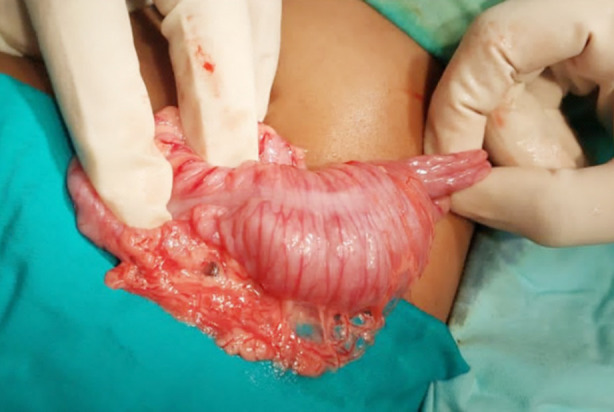
Ileocolic intussusception.

**Fig.2:**
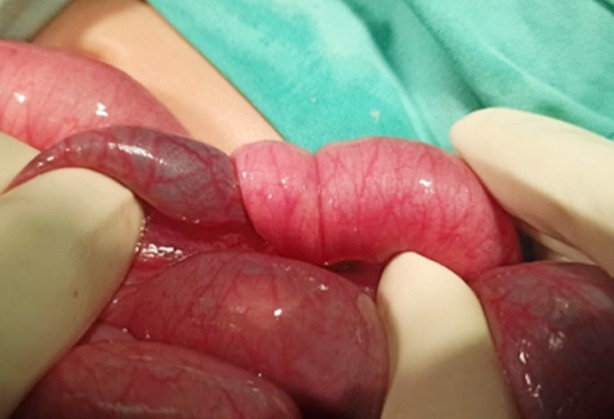
Postoperative ileoileal intussusception.

The seven months old female child presented to emergency room (ER) with three days history of intermittent colicky abdominal pain and excessive crying and two days history of bleeding per rectum. On examination she was dehydrated, listless with mild to moderate abdominal distension and currant jelly stool on digital rectal examination (DRE). Ultrasound confirmed the diagnosis of intussusception and the patient underwent laparotomy. Per operatively ileocolic intussusception was noted that was manually reduced successfully (Fig.1). Gut was hyperemic with few serosal tears but viable. No gross pathological lead points were noted and appendectomy was performed. Child was allowed orally on the 3^rd^ postop day after establishing bowel sounds, however she developed persistent vomiting. She was passing loose stool and had abdominal distension which was progressively increasing. Later the child developed episodes of excessive crying with in drawing of limbs. Nasogastric (NG) decompression was done and bilious output was noted. An ultrasound was done that stated “a concentric structure with alternating echogenic and hypo echogenic bands is seen in the right lower abdominal quadrant. Rest of the small bowel loops appear dilated, one of them measuring 20mm in diameter and showing ineffective peristalsis”. Xray erect abdomen and pelvis showed multiple air fluid levels. Hence decision to re explore was undertaken. At laparotomy, an ileoileal intussusception was found with proximal dilated gut loops. Fortunately the intessusception was manually reduced successfully with viable gut (Fig.2). Recovery was uneventful. No recurrence was noted at six months of follow up visits.

### Case-2

The six months old male child presented to ER with four day history of excessive crying with in drawing of limbs, vomiting, and bleeding per rectum since two days. At presentation, child was sick looking, dehydrated, had moderately distended abdomen and currant jelly stool in pamper. Ultrasound confirmed diagnosis and laparotomy was performed with perioperative findings of ileocolic intussusception that was manually reduced successfully after generously irrigating the gut walls with warm saline. Appendectomy was performed; no gross pathological lead points were noted. Post operatively the child was allowed orally after forty eight hours of observation however he developed vomiting that progressed to bilious. Nasogastric tube was passed which showed significant bilious output over the subsequent 48 hours. Child failed to pass stool and developed abdominal distension. Xray erect abdomen and pelvis showed air fluid levels with cut off sign and an ultrasound was done that reported intussusception. Patient underwent laparotomy and per operatively an ileoileal intussusception with gut necrosis was noted. Intestinal resection and anastomosis was performed. Histopathological evaluation of the specimen did not reveal any pathological lead point. Child followed an uneventful recovery with no recurrence.

### Case-3

The four months old male child presented to ER in toxic condition with five days history of excessive crying, vomiting and progressive abdominal distension with bleeding per rectum since two days. Diagnosis of intussusception was made by ultrasound and child underwent laparotomy after urgent resuscitation. Perioperative findings of ileocolic intussusception without a gross pathological lead point were noted, that was manually reduced successfully after copious gut irrigation with warm normal saline. Appendectomy was performed. Feeds were allowed on third postoperative day however the child developed episodic vomiting. He had mild abdominal distension that was initially attributed to postop ileus. Child was kept under observation for a further forty eight hours and then discharged as symptoms resolved and he was passing stool. He presented to the emergency room the next day with complaints of episodic abdominal pain with excessive crying and an episode of bilious vomiting. He had mild abdominal distension and passed loose stool on digital rectal stimulation.

An ultrasound was performed that showed intussusception in the left iliac fossa. As he was passing stool, we initially planned close observation. However he continued having excessive nasogastric tube output and persistent abdominal distension over the next 24 hours. Ultrasound was repeated that showed same findings and decision to perform laparotomy was undertaken. An ileoileal intussusception with multiple intergut loop adhesions was noted. Fortunately, gut was viable and manual reduction was successful. Child followed a satisfactory postop recovery.

## DISCUSSION

Postoperative intussusception (POI) is a rare cause of intestinal obstruction in children^4^ reported to follow many abdominal and non-abdominal operations^1^ but scarce literature reports are available on POI in patients operated primarily for intussusception.^2^ To the best of our knowledge, there are less than 50 reported cases thus making it a very rare entity. The diagnosis is elusive, particularly in the setting of recent surgery and thus requires a very high index of suspicion. The POI frequently presents with atypical prolonged ileus symptoms two to eight days following primary surgery with bilious vomiting being the most commonly reported presentation. Abdominal distension and increased bilious nasogastric tube output are other common presentations, with rare reporting of excessive crying, and bleeding per rectum with stool. None of the case studies in literature have yet reported a sausage shaped palpable abdominal mass among the signs (Table-I).

**Table-I:** Symptoms and Signs experienced by the studied patients.

	Case 1	Case 2	Case 3
Abdominal Pain	+	-	+
Abdominal distension	+	+	+
Vomiting	+	+	+
Increased nasogastric output	+	+	+
Bleeding per rectum	-	-	-
Passing stool	+	-	+
Sausage shaped mass	-	-	-

It was previously established that a contrast enema remains the gold standard investigation in intussusception as it has both diagnostic and therapeutic value however it is not readily available in all institutions and is contraindicated in patients with long duration of symptoms due to risk of perforation. Additionally, many institutions now avoid it due to undue radiation exposure. Hence ultrasound is currently the most commonly performed investigation and as evident from our case series, it can successfully diagnose a second intussusception.^5^

In cases where sonography is equivocal, expertise of the operator and sophistication of technique should be considered before disregarding the diagnosis. Small bowel intussusceptions are relatively difficult to diagnose as they are smaller than the ileocolic types, maybe found in varied location in the abdomen and frequently surrounded by many loops of dilated bowel because of obstruction. Once the POI is diagnosed, urgent surgical intervention should be sought as delayed treatment significantly increases mortality and morbidity.^1^ The mortality is reported to be as high as 6-7%.^4^ Among our patients, one had bowel necrosis at the time of exploration and had to undergo intestinal resection and anastomosis. Fortunately the recovery was satisfactory.

In the era of non-operative management many institutions are doing hydrostatic reduction of the primary intussusception by using ultrasound guidance.^6^,^7^ Ours is a tertiary care centre and in addition to catering to patients from within the city as well as the city’s suburbs, we also receive referrals from remote areas. Many of these patients have a delayed diagnosis, as they are usually treated at their local clinics initially and frequently misdiagnosed as dysentery. Hence, although we are practicing non operative management by attempting ultrasound guided hydrostatic reductions, most of the cases that we receive are candidates for surgical intervention due to delayed presentation and risk of complications.^8^ We acknowledge that all patients of our series underwent lapratomy without attempts at hyrostatic reduction as they had presented in sick condition with symptom duration of more than 72 hours. We do minimally invasive surgery in selected patients when the expertise is available but most are managed by open surgery.

Several theories have been proposed to understand the pathophysiology of POI. These include early adhesions, excessive bowel manipulation and desiccation, altered peristalsis, neurogenic factors, electrolyte imbalances, and drugs (anaesthetics and opioids).^9^ It is debatable that non operative reduction of primary intussusception may reduce the occurrence of POI due to minimal tissue handling that is inevitable with open surgery. Currently, suggested preventative measures for POI include gentle handling, avoidance of desiccation of the bowel and using a minimally invasive approach although POI has also been reported after laparoscopic surgeries. More research is needed in this area to obtain a better understanding.

## CONCLUSION

POI is a rare cause of intestinal obstruction with POI after surgical reduction of ileocolic intussusception being an extremely rare variant. The diagnosis needs a very high index of suspicion and should be considered in patients presenting with atypical prolonged ileus. Abdominal ultrasonography is diagnostic in the majority of cases and early surgical intervention is warranted to reduce mortality and morbidity.

### Authors Contribution:

**MAC** and **C:** Concept and Design of study.

**C, AJ** and **SAB:** Acquisition and Interpretation of data.

**C:** Final Editing of Manuscript.

**MAC:** Supervision, responsible for integrity and accuracy of work, review, final approval.
